# Color Can Shorten Breakthrough Times in Continuous Flash Suppression through Increased Salience and Task Relevance

**DOI:** 10.3390/vision5010013

**Published:** 2021-03-16

**Authors:** Christian Valuch

**Affiliations:** Department of Experimental Psychology, University of Goettingen, 37073 Goettingen, Germany; christian.valuch@psych.uni-goettingen.de

**Keywords:** interocular suppression, consciousness, color vision, visual search, attentional templates, early visual system, awareness, continuous flash suppression, binocular rivalry

## Abstract

Color can enhance the perception of relevant stimuli by increasing their salience and guiding visual search towards stimuli that match a task-relevant color. Using Continuous Flash Suppression (CFS), the current study investigated whether color facilitates the discrimination of targets that are difficult to perceive due to interocular suppression. Gabor patterns of two or four cycles per degree (cpd) were shown as targets to the non-dominant eye of human participants. CFS masks were presented at a rate of 10 Hz to the dominant eye, and participants had the task to report the target’s orientation as soon as they could discriminate it. The 2-cpd targets were robustly suppressed and resulted in much longer response times compared to 4-cpd targets. Moreover, only for 2-cpd targets, two color-related effects were evident. First, in trials where targets and CFS masks had different colors, targets were reported faster than in trials where targets and CFS masks had the same color. Second, targets with a known color, either cyan or yellow, were reported earlier than targets whose color was randomly cyan or yellow. The results suggest that the targets’ entry to consciousness may have been speeded by color-mediated effects relating to increased (bottom-up) salience and (top-down) task relevance.

## 1. Introduction

### 1.1. Background

Color vision allows us to discern most broad-band natural object reflectance spectra [1,2] and probably evolved because it provided critical survival benefits in foraging [3] and social [4] situations. Local color contrasts increase the bottom-up salience of an object [5,6], which in turn attracts spatial attention to the object’s location, resulting in prioritized neurocognitive processing of the attended information [7]. In everyday environments, local color contrasts are usually associated with interesting and often relevant objects which can grab attention in a bottom-up manner, such as red light or a stop sign at an intersection [5,7]. However, color can also be used for directing attention in a top-down way [8,9], such as when looking for friends in a crowd of people and expecting them to wear clothes of a particular color. While it is well established that color is a powerful feature for guiding selective visual attention, it is not entirely clear through which mechanisms color affects how quickly an object is consciously perceived [10]. A traditional view of the relationship between attention and consciousness posits that attention acts as a ‘gatekeeper for consciousness’, that is, that the selection of a stimulus by attention precedes conscious perception [11,12,13]. However, attention and consciousness are distinct processes that are not always aligned [14], which is why top-down attention does not necessarily entail conscious processing [10,15].

To investigate the factors involved in conscious perception and to understand which types of processing occur unconsciously, a range of perceptual suppression techniques has been developed to render visual stimuli temporarily invisible [16,17,18]. Continuous Flash Suppression (CFS) is a relatively new technique which provided numerous insights into the factors that influence consciousness [19,20,21]. CFS builds upon the binocular rivalry (BR) phenomenon that occurs when the same participant’s left and right eye is presented with different, incompatible images [22,23,24]. Under such dichoptic conditions, conscious perception usually settles on one of these rival images while the other image remains invisible or unconscious due to interocular suppression. In classical BR, perception cycles through distinct phases of perceptual dominance of one or the other stimulus, with a perceptual switch usually occurring every few seconds [23,25]. CFS is a particular version of BR in which one is presented with a high-contrast pattern stimulus that is updated over time, often at a frequency of 10 Hz [21,24]. The other eye is presented with a relatively static and usually smaller target stimulus. The steep difference in stimulus strength between these two eyes’ inputs ensures that the CFS pattern mask is always perceived at the beginning of each experimental trial [23], and it usually takes several seconds until the initially invisible target stimulus can be reported by the participants [10,20].

### 1.2. The Present Study

The present study used a “breaking CFS” procedure, in which the *breakthrough time* denotes the time it takes participants to report an (initially invisible) target stimulus. The goal was to investigate how breakthrough time is influenced by the color contrast between the relevant target stimuli and the CFS masks on the one hand, and the task relevance of a specific color on the other hand. To that end, the current study used oriented gratings as targets and asked participants to report the orientation as soon as they could perceive it. The relevant targets were presented in of two possible colors (cyan or yellow). Typical CFS pattern masks were used to delay perception of the targets through interocular suppression. The (bottom-up) color contrast between the targets and the CFS masks was manipulated by either using the same color for the target and the CFS masks (resulting in a low color contrast), or different colors for the target and the CFS masks (resulting in a high color contrast). To investigate whether color can also have top-down effects on breakthrough times [10], the present study also manipulated the color relevance for the discrimination task. In one block of experimental trials, color was irrelevant. Here, only one grating was presented in each trial, and the color of this target grating could change randomly from trial to trial. The time it took for the targets to break suppression was compared to another block of experimental trials, in which color was relevant. Here, participants were always presented with two gratings of different colors, and needed to report the grating with a specific task-relevant color.

Since the targets randomly appeared either above or below the fixation point, the participants had to perform a visual search through the potential target locations. In the color-irrelevant block, only one grating was presented, making the task arguably relatively easy, as it did not require a decision about whether a grating was the target, or not. Participants could simply report the orientation of any grating they saw. However, the lack of knowledge about how the target will look might be disadvantageous, because it prevented participants from tuning their search to a specific target color. After all, the targets competed with the strong CFS mask, making the search task more difficult than it might appear at first glance. Here, color might still help participants to localize the target in trials where the target and CFS mask colors differed, due to the increased color salience which might attract spatial attention to the target’s location in a bottom-up manner [6,7]. Conversely, in the color-relevant block, participants were presented with two gratings of different colors in every trial, which arguably made the task more complex because it now required to reject gratings with the task-irrelevant color and only respond to gratings with the task-relevant color. However, this condition also allowed participants to specifically tune their visual search to the task-relevant color. Knowing what target-color to look for should help them direct spatial attention towards stimuli that matched this search template and, at the same time, avoid directing attention to stimuli that did not match this template [8,9]. Therefore, the color-relevant block should maximize the top-down effects of prior knowledge due to the combined effect of facilitated spatial detection of targets and rejection of non-targets based on color.

The hypotheses for the different conditions in the present study were the following. First, suppose color leads to faster target reports due to increased bottom-up salience. In that case, the breakthrough time should be shorter in trials in which the colors of the target and the CFS masks were different (i.e., where the color contrast was high). Conversely, the breakthrough time should be longer in trials in which the target and CFS mask had the same color (i.e., where the color contrast was low). Second, suppose a specific color is relevant for solving the task, and participants can focus on this relevant color to detect and report stimuli that match this color faster. In that case, targets should be reported faster in conditions where target color is relevant compared to conditions where target color is irrelevant (and target color could change from trial to trial). To sum up, participants either searched for a target of a particular color or not, and these targets were either the same color as the mask or not. This allowed for simultaneously measuring the influence of sensory (contrast) and task-based (relevance) influences of color on breakthrough times in CFS.

To verify whether color-related effects on breakthrough times differ depending on how robustly the perception of the targets is suppressed by CFS, the present study also manipulated the spatial frequency (SF) of the targets. Previous research demonstrated that typical CFS patterns could robustly suppress gratings with a relatively low spatial frequency (LSF) [26,27]. In contrast, perception of gratings with a high spatial frequency (HSF) is usually less perturbed by the same CFS patterns. This effect is probably at least partly due to the fact that traditional CFS pattern masks are also dominanted by low SF components, which interfere more strongly with perceptual processing of low SF targets [26]. Hence, the present study included HSF gratings to check if any color-related effects occured independently of whether perception of the gratings are robustly suppressed, or not. Thus, testing for interactions between the SF factor and the factors of color relevance and color contrast should inform whether the color-related factors primarily affect breakthrough times in conditions in which the perception of the targets is robustly delayed. In addition, testing the interaction between the factors of color contrast and color relevance should reveal whether the sensory benefits of high color contrast could be modulated by color relevance, such that, for example, the effect of color salience would be diminished once a specific color becomes relevant for the task.

## 2. Materials and Methods

### 2.1. Participants

A total of 40 students from Goettingen participated in return for partial course credit or financial compensation. All participants were pre-screened for intact or fully-corrected visual acuity and color perception, as well as their ability to fuse the frames of the dichoptic stimuli into one stable percept, when viewed through the mirror stereoscope. Any recruited participant who failed in one of these initial screening tests was excluded from participation in the experiment. The decision on the final sample size was based on a priori considerations. A power analysis (assuming medium-sized effects of *f* = 0.25 for color contrast and color relevance, a power of 1 − = 0.80, and a correlation of *r* = 0.5 among repeated measures) suggested a minimum sample size of 18 participants. Due to the possibility that the actual effects or correlations might be smaller and to allow for full counterbalancing of task order and target colors, the planned sample size was set to 40, which should thus be sufficient to detect the hypothesized effects.

### 2.2. Apparatus

Visual stimuli were presented on a 22-in. color CRT monitor (ViewSonic PF817). The screen resolution was set to 1024 × 768 pixels with a vertical refresh rate of 140 Hz. The viewing distance was fixed at 57 cm by means of a chin and forehead support, on which a mirror stereoscope was mounted. Dichoptic stimulation was realized by presenting different stimuli side-by-side at peripheral screen locations and viewing the screen through the stereoscope such that each of the rivalrous stimuli was delivered to one eye only. Participants reported the target stimuli manually using the left and right “control” keys on a standard USB keyboard. The experiment was implemented using the software Presentation (Neurobehavioral Systems, Berkeley, CA, USA) and run under Windows 7 on a PC with an Intel Core i7 2600 CPU and 16 GB of RAM. The luminance of the colored stimuli was measured using a PR-524 LiteMate photometer (Photo Research Inc., Chatsworth, CA, USA) to ensure that the two alternative colors (cyan vs. yellow) were isoluminant.

### 2.3. Stimuli

**Figure 1:**
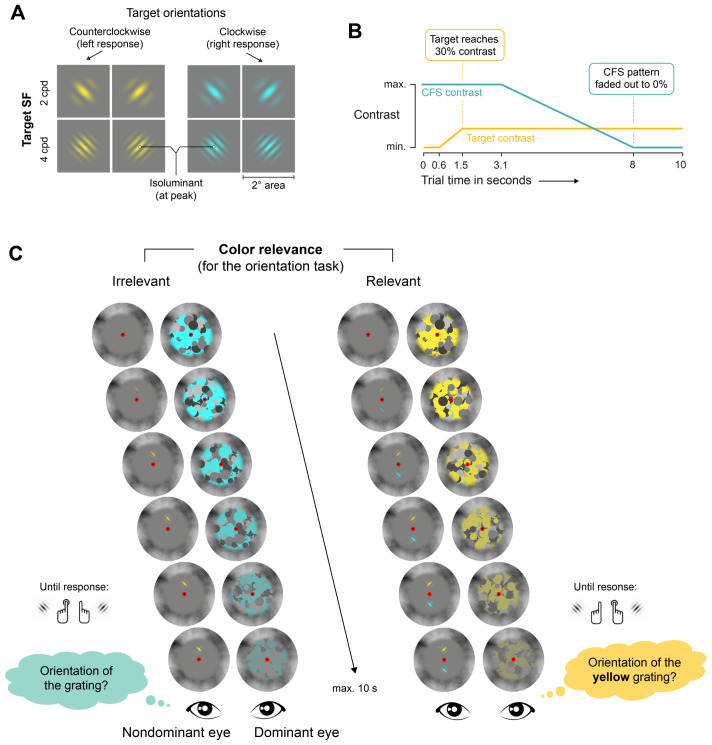
(**A**) Targets were counterlockwise or clockwise rotated Gabor gratings presented above or below the fixation point. Targets had a SF of 2 cpd or 4 cpd and were rendered either in yellow or cyan. Colors were isoluminant at their peak luminance values. (**B**) The target grating’s contrast was ramped up in the first phase of the trial, while the contrast of the CFS masks decreased over time in the second phase of the trial. The trials had a maximum duration of 10 s but ended as soon as the participants gave a response. (**C**) The targets were delivered to the non-dominant eye using a mirror stereoscope. Perception of the targets was interocularly suppressed by presenting a random sequence of CFS masks with a 10 Hz update frequency to the dominant eye. The targets had either the same or different color as the CFS masks. The color of the CFS masks varied randomly between cyan and yellow from trial to trial. In each trial, the participants’ task was to report the target’s orientation using key presses as soon as they could perceive it. As soon as the participants responded, the trial ended. Left: in the color irrelevant block, only one grating was presented in each trial, which varied randomly between cyan and yellow from one trial to the next. Right: In the color relevant block, two gratings were presented in each trial, and participants reported gratings that matched the instructed target color (either yellow or cyan).

Dichoptic stimuli were displayed on a neutral gray background (27.7 cd/m${}^{2}$) within circular apertures with a diameter of 9${}^{\circ }$ to facilitate stable vergence. The apertures had a 1/f noise pattern at their outer border, which extended about 2${}^{\circ }$ into the center, where it smoothly faded into the gray background. Screen areas outside the circular apertures were set to black (0.1 cd/m${}^{2}$). A red fixation oval with a diameter of 0.5${}^{\circ }$ and a small black dot (0.1${}^{\circ }$) in the center were presented throughout the experiment as a fixation point. The participants were instructed to fixate on this dot with their eyes throughout the experiment. The target stimuli (see Figure 1A) were colored sinusoidal gratings covering a square region of 2${}^{\circ }$, that were masked with a circular Gaussian window (*SD* = 0.25${}^{\circ }$). The targets were always presented to the non-dominant eye at a center-to-center distance of 1.5${}^{\circ }$ above or below the central fixation point. Targets varied in color (cyan vs. yellow), orientation (45${}^{\circ }$ counterclockwise or 45${}^{\circ }$ clockwise with repect to a vertical orientation) and spatial frequency (2 vs. 4 cycles per degree; cpd). The maximum contrast of the targets was set to 30% to ensure robust suppression effects.

Perception of the targets was delayed by presenting high-contrast CFS pattern masks to the participants’ dominant eye, which were updated at a rate of 10 Hz. A set of CFS patterns was pre-generated using MATLAB (MathWorks, Natick, MA, USA) to standardize the visual properties of the different CFS patterns. The CFS patterns consisted of randomly arranged and partly overlapping filled circles, with diameters ranging from 0.5${}^{\circ }$ to 1.7${}^{\circ }$ and different gray and color values. A goal characteristic of the CFS patterns was that about half of all pixels in each mask should have one of three gray values (dark gray: 6.1 cd/m${}^{2}$; medium gray: 27.7 cd/m${}^{2}$, light gray: 67.3 cd/m${}^{2}$), whereas the other half of all pixels should have one of the color values (yellow with RGB values of 250/227/60 and a luminance of 100.0 cd/m${}^{2}$ and cyan with RGB values of 72/255/255 and a luminance of 100.1 cd/m${}^{2}$). From a large number of randomly generated CFS patterns, 100 patterns were selected that fulfilled these criteria most closely. Two versions of each pre-selected CFS pattern were generated (one for each target color). This minimized the possibility that CFS patterns could be more effective in one of the conditions due to a particular spatial configuration of the elements of the patterns.

### 2.4. Procedure and Design

#### 2.4.1. Eye Dominance Assessment

At the beginning of each experimental session, the stereoscope was calibrated for each participant to achieve stable binocular fusion and it was verified that each eye’s stimulus frame was fully visible through the respective mirror’s field of view. Since it influences the strength of interocular suppression whether the CFS masks are presented to the dominant eye or the non-dominant eye [28], the present study tried to keep this factor constant across participants. It is essential to distinguish between sighting dominance and sensory dominance, which often do not overlap and need to be determined using different procedures. Specifically, sensory dominance is critical for CFS’s strength and should be measured under conditions that are as close as possible to the experimental context [29]. Therefore, eye dominance was assessed using a short screening experiment [21,28]. Each participant completed 60 trials with CFS masks presented to either the left or right eye and an arrow as a target presented to the other eye. CFS masks appeared at 10 Hz and were very similar to the ones used in the present study’s main experiment, except that they were achromatic and also contained black and white circle elements. A target arrow (4.1${}^{\circ }$× 1.4 ${}^{\circ }$) appeared ±1.7${}^{\circ }$ above or below a central fixation point. The contrast of the arrow was increased from 0% to 100% over 5 s. Simultaneously, the contrast of the mask decreased from 100% to 0%. All stimuli were presented in the same circular frames and with the same neutral gray background as in the main experiment. Participants reported the arrow direction by pressing the left or right control key. Trials ended by pressing one of the response buttons or after a maximum duration of 10 s. The dominant eye was defined as the eye with a longer suppression duration when the CFS masks were presented to this eye.

#### 2.4.2. Trial Procedure

During the experiment, CFS masks were always presented to the dominant eye, whereas targets were presented to the non-dominant eye (see Figure 1). Each trial of the main experiment started with the presentations of CFS masks to the dominant eye at a rate of 10 Hz. The onset of the targets occurred at 0.5 s into the trial. Target contrast increased from 0 to 30% over a period of 1 s and remained constant at 30% until the trial ended (see Figure 1B). This contrast ramp was implemented to avoid abrupt onsets of the target, which might capture attention and break suppression instantly. Participants were instructed to report the orientation of the targets as soon as possible. After 3 s into the trial, the contrast of the CFS masks gradually decreased to zero over a period of 5 s. This reduction of CFS contrast was included to allow all participants to eventually recognize the target, which otherwise might not be the case, as individual differences in interocular suppression are large and suppression could last longer than the maximum trial duration for some participants [21,25]. After reaching the lowest contrast level of the CFS masks, only the medium gray stimulus background was presented to the participant’s dominant eye for the remainder of the trial. Trials ended immediately after participants gave a response or after a maximum duration of 10 s.

#### 2.4.3. Task

The participants’ task required to press the left “control” key using the left index finger, if the target grating was tilted 45${}^{\circ }$ counterclockwise (from the vertical), and to press the right “control” key with the right index finger, if the target grating was tilted 45${}^{\circ }$ clockwise. If participants gave an incorrect response (e.g., if they pressed the left “control” key when the target was in fact tilted clockwise), they were played a brief error tone (430 Hz, for a duration of 150 ms). If they did not respond before the maximum trial duration of 10 s, they also received this error feedback. The start of the next trial was briefly delayed whenever the participants received the error feedback. Otherwise, if participants gave the correct response, the next trial started after a fixed inter-trial interval of 1 s.

#### 2.4.4. Experimental Design

Every participant was measured in all experimental conditions in a full factorial 2 (Target SF: 2 cpd vs. 4 cpd) × 2 (Target/CFS color: different vs. same) × 2 (Color relevance: irrelevant vs. relevant) design. Each participant completed 768 experimental trials, divided into two blocks of 384 trials. Color relevance varied between blocks and the order of the conditions (relevant→irrelevant or irrelevant→relevant) was counterbalanced across participants. Within each block, all combinations of grating and CFS mask colors, target positions (above or below fixation), orientations (counterclockwise vs. clockwise) and SF (low vs. high) occurred with equal frequency and varied randomly from trial to trial.

In the *color irrelevant* block, only one grating was presented to the non-dominant eye (see Figure 1C). The grating’s location (above or below the fixation point) was randomized across trials. Participants reported this gratings’ orientation irrespective of its color. In half of the trials, the target had the same color as the CFS mask, in the other half of trials, the target had the different color. In the *color relevant* block, the procedure was identical, only that two gratings were presented in each trial, and these two gratings always had different colors. Here, the assignment of color to location was randomized across trials. Importantly, participants were instructed to report the orientation of a grating with a particular color (e.g., ‘yellow’) throughout the block and ignore gratings of the other color, which served as a distractor. In half of the trials, the orientation of the distractor grating was the same as the orientation of the target grating, in the other half of the trials, the orientation of the distractor grating was different than the target grating. The instructed target color was counterbalanced across participants. In a given trial, target and distractor grating always had the same SF.

After completing runs of 64 trials in one go, participants were shown a pause message (e.g., “Pause—part 1 of 6 completed”). Participants were instructed to use these pauses to rest briefly and press the “enter” key to continue with the experiment as soon as they were ready. At the outset of each task block, participants received written instructions and underwent a training block consisting of 32 trials, which allowed them to get acquainted with the task and the experimental stimuli.

## 3. Results

### 3.1. Description of the Dataset

The statistical analysis was conducted using R [30]. The full dataset encompassed 30,720 trials (768 trials for each of the 40 participants). In 15 trials (0.05% of the data), participants failed to respond within the maximum trial duration of 10 s. These trials were excluded from all further analyses. In a total 1059 trials, participants, made errors. The average error rate across participants was 3.4% (ranging from 0.4% to 20.2%, although only two out of 40 participants made errors in more than 10% of the trials). On average, in 4.7% (*SE* = 1.87) of trials with correct responses, participants responded before the target had reached its final contrast. In 45.7% (*SE* = 3.43) of trials, participants responded after the target had reached its final contrast and while the mask also still had the full contrast. In 38.5% (*SE* = 3.03) of trials, the response came after the mask started to gradually loose contrast but before it was fully faded out. Finally, in 11.1% (*SE* = 2.62) of trials, participants responded after the mask was faded out but before the trial ended. Table 1 presents the descriptive statistics for breakthrough time and error rate for the eight experimental conditions in the present study.

### 3.2. Breakthrough Time

#### 3.2.1. Main Analysis of Experimental Effects

**Figure 2:**
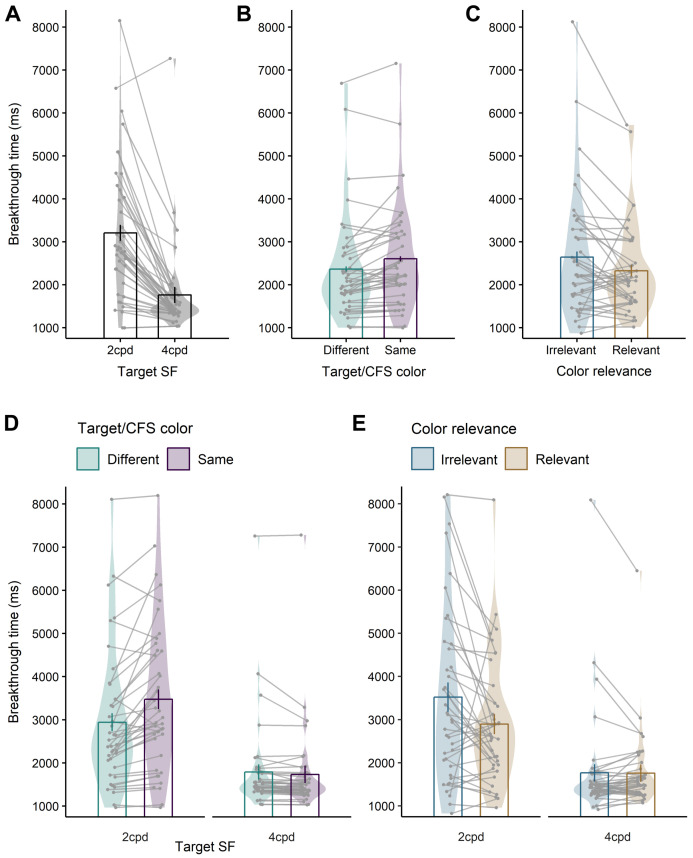
Significant results of the repeated measures ANOVA of breakthrough times in trials with correct responses. The data points in each plot’s background represent the medians of the breakthrough time of individual participants, which were entered into the statistical model. Lines connect data points belonging to the same participants. The violin plots represent the distribution of data within each condition, and the bar graphs represent the condition means. Error bars represent ±1.96 *SE* after correcting for between-participants variance [31]. (**A**) Main effect of target SF. (**B**) Main effect of target/CFS color. (**C**) Main effect of color relevance. (**D**) Interaction of Target SF × Target/CFS color. (**E**) Interaction of Target SF × Color relevance.

The statistical analyses of breakthrough time were based on a total of 29,646 trials with correct responses (or 96.5% of the presented trials). The median breakthrough time in milliseconds for each participant and condition was entered into a repeated measures ANOVA with the within-participant factors *target SF* (2 cpd vs. 4 cpd), *target/CFS color* (same color vs. different color), and *color relevance* (relevant vs. irrelevant). The significant effects of this analysis are plotted in Figure 2.

As expected, the breakthrough times yielded a significant main effect of target SF, *F*(1, 39) = 57.9, *p* < 0.001, $\eta_{p}^{2}$ = 0.60, with robust and long suppression durations for LSF targets of 2 cpd (*M* = 3209 ms, *SE* = 252.5), and rather ineffective suppression, resulting in much shorter breakthrough times for HSF targets of 4 cpd (*M* = 1762 ms, *SE* = 165.6). Furthermore, the analysis revealed a main effect of target/CFS color, *F*(1, 39) = 15.1, *p* < 0.001, $\eta_{p}^{2}$ = 0.28, with, on average, shorter breakthrough times when the target and the CFS mask had different colors (*M* = 2366 ms, *SE* = 190.5) compared to when they had the same color (*M* = 2604 ms, *SE* = 196.6). The analysis also yielded a main effect of color relevance, *F*(1, 39) = 5.5, *p* = 0.024, $\eta_{p}^{2}$ = 0.12, with shortened breakthrough times when participants searched for a specific color (*M* = 2327 ms, *SE* = 167.1) compared to when target color was irrelevant and could change from trial to trial (*M* = 2643 ms, *SE* = 232.7).

The analysis revealed a significant interaction of Target SF × Target/CFS color, *F*(1, 39) = 27.1, *p* < 0.001, $\eta_{p}^{2}$ = 0.41. For LSF targets, post-hoc tests showed that in trials where targets and CFS masks were differently colored, the suppression durations were shorter (different colors: *M* = 2942 ms, *SE* = 247.2; same color: *M* = 3475 ms, *SE* = 270.1; *t*[39] = –4.6, *p* < 0.001). In contrast, for HSF targets, there was a slight, albeit statistically signficant, opposite tendency, resulting in *longer* suppression durations when targets and CFS masks were differently colored (different colors: *M* = 1789 ms, *SE* = 170.0; vs. same color: *M* = 1734 ms, *SE* = 162.1; *t*[39] = 2.1, *p* = 0.047). However, this slight opposite tendency might not reflect a robust and genuine effect, as it was not statistically significant in a control analysis that excluded participants with particularly short or long breakthrough times (see Supplementary Materials, Analysis S2).

The analysis also revealed an interaction of Target SF × Color relevance, *F*(1, 39) = 9.0, *p* = 0.005, $\eta_{p}^{2}$ = 0.19. Follow-up t-tests showed that the effect of task was significant for LSF targets (color relevant: *M* = 2896 ms, *SE* = 228.2; vs. color irrelevant: *M* = 3521 ms, *SE* = 318.2, *t*[39] = –2.7, *p* = 0.009) but not for HSF targets (color relevant: *M* = 1758 ms, *SE* = 139.9; vs. color irrelevant: *M* = 1766 ms, *SE* = 195.0, *t*[39] = −0.1, *p* = 0.918).

There was no interaction of Color relevance × Target/CFS color, *F*(1, 39) = 0.6, *p* = 0.435, suggesting that the influence color contrast was independent of whether participants searched for a specific target color. There was also no sign of a three-way interaction of Color relevance × Target/CFS color × Target SF, *F*(1, 39) = 1.2, *p* = 0.279.

Breakthrough times in LSF conditions were much higher compared to HSF conditions, and the absence of color-related effects for HSF targets could be related to the more constrained variance in these conditions. To allow a fair comparison in such situations, a simple normalization method was proposed in previous work [32]. To this end, breakthrough time in each trial was divided by the average breakthrough time of the respective SF condition. This control analysis replicated both interactions from the main analysis, hence excluding the possibility that the absence of color-related effects with HSF gratings could be a statistical artifact due to the absolute difference in breakthrough times between SF conditions (see Supplementary Materials, Analysis S1).

#### 3.2.2. Role of the Distractor for the Task Effect

Apart from the task instructions, a potentially critical difference was that two gratings were presented in the color-relevant conditions whereas only one grating was presented in the color-irrelevant conditions. Accordingly, one of the gratings (either the target or the distractor) had a different color than the CFS masks in every trial of the color-relevant conditions. In contrast, the target/CFS color was different only in every other trial in the color-irrelevant conditions. Therefore, it is important to look closer if this difference might explain the effect of color relevance with LSF targets.

First, we can compare breakthrough time in trials where targets and CFS masks had different colors. This way, the color difference between target and CFS mask was constant between the different task contexts. This confirmed the task-related effect of color relevance, with shorter breakthrough times when the target color was relevant (*M* = 2675 ms, *SE* = 216.1) compared to when the target color was irrelevant (*M* = 3209 ms, *SE* = 312.2), *t*(39) = –2.5, *p* = 0.015. Second, we can compare conditions in which targets and CFS masks had the same color. Here, indeed a potentially critical difference exists between color-relevant and irrelevant conditions because the relevant conditions included a distractor that had a different color than the CFS mask, and this could also contribute to a shortening of breakthrough times. Again, the effect of color relevance was confirmed, with shorter breakthrough times when the target color was relevant (*M* = 3117 ms, *SE* = 257.5) compared to when the target color was irrelevant (*M* = 3832 ms, *SE* = 344.2), *t*(39) = –2.6, *p* = 0.014. The slightly larger difference in breakthrough times in the second comparison suggests that the salient distractor may have contributed to the shorter breakthrough times in the color-relevant trials. However, comparing the task-related effects statistically between the target/CFS color conditions showed that no significant difference in the size of task-related color relevance effect existed, *t*(39) = 0.95, *p* = 0.347. To sum up, a closer look at the data suggests that the distractor’s presence alone cannot easily explain the task-related effect of color relevance.

### 3.3. Error Rates

It is also crucial to check if the observed differences in breakthrough times could have resulted from a speed-accuracy tradeoff. For example, in conditions where the targets are more difficult to discriminate, participants could have chosen a more cautious response strategy and deliberately take more time to avoid errors. If a speed-accuracy tradeoff could explain the effects in breakthrough times, there should be higher error rates in conditions where breakthrough times are shorter and lower error rates in conditions where breakthrough times are longer. To check this possibility, a full analysis of the arcsine transformed error rates was performed for all conditions.

The analysis of arcsine transformed error rates yielded a significant interaction of Target SF × Color relevance, *F*(1, 39) = 7.9, *p* = 0.008, $\eta_{p}^{2}$ = 0.17. For LSF targets, error rate did not differ depending on whether target color was relevant (*M* = 0.16, *SE* = 0.02) or irrelevant (*M* = 0.16, *SE* = 0.02), *t*(39) = –0.53, *p* = 0.599. For HSF targets, error rate was higher when target color was relevant (*M* = 0.16, *SE* = 0.01) compared to when it was irrelevant (*M* = 0.12, *SE* = 0.01), *t*(39) = 3.3, *p* = 0.002. The analysis also revealed a second interaction of Color relevance × Target/CFS color, *F*(1, 39) = 14.5, *p* < 0.001, $\eta_{p}^{2}$ = 0.27. When target color was relevant for the task, error rate was lower if targets and CFS masks had different colors (*M* = 0.14, *SE* = 0.01) compared to when they had the same color (*M* = 0.18, *SE* = 0.02), *t*(39) = –3.8, *p* < 0.001. When color was irrelevant, error rate did not differ depending on whether targets and CFS masks had the same (*M* = 0.13, *SE* = 0.02) or different colors (*M* = 0.15, *SE* = 0.02), *t*(39) = 1.6, *p* = 0.128. No other effects were statistically significant in the analysis of error rates. To sum up, while error rates were also partially influenced by the experimental manipulations, there are no indications that differences in breakthrough times could be explained by a speed-accuracy tradeoff.

## 4. Discussion

The current study investigated whether and how color facilitates perception of relevant target stimuli that are difficult to discriminate due to interocular suppression. Using CFS, the perception of target stimuli presented to the non-dominant eye was delayed by presenting dynamic visual patterns to the dominant eye [19,20,21]. The efficacy of CFS was manipulated by using either LSF targets (which are typically robustly suppressed by CFS) or HSF targets (which typically remain relatively unperturbed by CFS [26]). Consistent with previous research, LSF targets were robustly suppressed from perception, which was not the case for HSF targets [26,27]. Notably, facilitative effects of color consistently shortened breakthrough times for LSF targets. In contrast, no consistent color-related effects occurred with HSF targets that were not robustly suppressed by the present study’s CFS masks.

The robust suppression of LSF gratings compared to HSF gratings confirmed the predictions based on previous literature [26]. At least three reasons could underlie this effect. First, the Mondrian patterns typically used in CFS studies are characterized by a 1/f frequency spectrum in which lower SFs are more dominantly represented. Thus, the stronger similarity in the SF profile between LSF gratings and CFS masks could result in a feature-selective suppression effect [26,33]. This is supported by the observation that gratings with higher SFs are better suppressed by filtered CFS masks in which higher SFs are emphasized [26,27]. Second, classical BR studies with static gratings also showed that suppression durations are prolonged for LSF compared to HSF gratings [34]. Since CFS is a variant of BR, it is not surprising that interocular suppression by CFS is also most robust for LSF stimuli. Third, binocular summation could contribute to the better visibility of HSF gratings compared to LSF gratings. More specifically, the dynamic updating of the CFS masks (with a new pattern configuration every 100 ms) could, at times, result in temporarily decreased masking strength. For example, parts of a (yellow) mask could contain homogenously gray areas. If these areas correspond with the location of a yellow target, the contrast of the target against the fully gray background (from both eyes) could be increased relative to other points in time where the CFS pattern contains yellow patches at the positions corresponding with the location of the yellow target. This temporary facilitation might be more pronounced for HSF gratings, since the number of bars that make up the target is higher, which could allow easier recognizability of the target, even if only part of it would have a higher contrast (relative to the mask image) (Whether the difference in the number of available diagnostic features (visible bars) in LSF vs. HSF gratings contributes significantly to lower visibility of LSF gratings could be specifically investigated in future research. For example, the size and slope of the Gaussian window could be manipulated so that the number of visible bars is comparable between LSF and HSF gratings. I thank an anonymous reviewer for pointing out this alternative explanation for the difference between LSF and HSF gratings, as well as the suggestion on how to address it in further research.). Regardless of which of these mechanisms was mainly responsible for the observed effects in the present study, the suppression of LSF gratings was much more robust, resulting in overall prolonged breakthrough times.

The reportability of the robustly suppressed LSF gratings benefitted from two color-related effects. First, color led to shorter breakthrough times through a bottom-up (or sensory) benefit [6,7]. When the targets and the CFS masks had different colors (that is, when the color contrast was high), the initially invisible targets were reported faster than when the targets and CFS masks had the same color (that is, when the color contrast was low). The size of this effect was independent of whether or not participants searched for a specific color. Second, it generally mattered whether participants searched for *one* particular target color, or not. When target color was task relevant, for example, when participants always reported the yellow gratings’ orientation but ignored cyan gratings, the yellow targets were reported significantly faster compared to trials where target color was irrelevant and could change from trial to trial. This confirms that color can also facilitate perception in a top-down way, if it is relevant for the task. These two main findings will now be discussed in some more detail.

### 4.1. The Bottom-Up Effect of Color Salience on Breakthrough Times

The bottom-up effect of target/CFS color contrast was robust and independent of whether participants searched for one specific color. Previous research on the CFS method showed, to some extent, that the similarity between the features of the suppressed stimulus and the CFS mask affects the strength of suppression. For example, previous research reported that the degree to which CFS suppresses oriented gratings could depend on orientation similarity: Gratings with cardinal orientations are more robustly suppressed by traditional Mondrian patterns, which primarily consist of cardinal orientations (vertical and horizontal edges), whereas gratings with oblique orientations can escape suppression by the same Mondrian patterns more easily [26]. Related to this, BR experiments also showed that interocular suppression is stronger when the orientations of competing gratings are similar [33]. Also in line with the view that feature similarity modulates the strength of interocular suppression, another study found that moving stimuli are better suppressed by moving Mondrian patterns than traditional Mondrian patterns with a discrete update frequency [35]. Regarding color, however, the results so far have been less clear. While one study suggested that it does not matter whether achromatic or colorful CFS masks are used in combination with achromatic targets [26], a subsequent study reported that achromatic targets are more robustly suppressed when the CFS masks contain a range of different colors, in comparison to CFS masks that contain different gray values only [36]. However, if color relationships between targets and CFS masks modulate suppression strength was not yet investigated.

The present study clarifies the open question of whether feature-selective suppression also occurs for colors during CFS by explicitly manipulating the similarity or contrast between the targets’ and CFS masks’ color. The present data leaves barely any doubt that in CFS, feature-selective suppression also occurs for the color dimension of the rivaling stimuli. This bottom-up effect is robust and occurs independent of the task relevance of color, suggesting that it is situated at an early, presumably sensory level of the visual processing hierarchy, where it directly affects the competition between the different eyes’ stimulus signals. Previous studies on binocular rivalry and CFS often focused on luminance contrast as a central stimulus-dependent determinant of interocular suppression. Typically, increasing the luminance contrast of one stimulus will increase its perceptual predominance and likelihood to break interocular suppression [21,22,23]. The results of the current study suggest that such contrast effects are not limited to the luminance dimension. Noteworthy, the luminance of the rivalrous stimuli in the present study was kept constant, but the color contrast was manipulated by using targets of either the same or different colors as the CFS masks. The neural processing of a relevant stimulus at early levels of the visual processing hierarchy and the timing of when this stimulus enters consciousness might thus generally benefit from a stronger feature salience, regardless of the exact feature dimension [7]. Strong contrasts in the neural representations between the irrelevant CFS mask and the relevant target might represent a good signal-to-noise ratio in terms of neural processing of the sensory signals. In contrast, with low contrast, the resulting signal-to-noise ratio would be poor.

The result that targets which differed in color from the CFS masks were detected faster regardless of whether color was relevant for solving the task complements recent findings, which have shown that visual working memory (VWM) and color salience with respect to other objects can independently influence breakthrough times [37]. Unlike in the current study, where salience was established by the difference between the target and the CFS mask, this previous previous study defined salience as the contrast between a target and other distractors, which were all presented to the same eye (while achromatic CFS masks were presented to the other eye). Breakthrough times were shorter when the targets were distinct from all distractors but also when the targets matched the search instructions in terms of color. A race model analysis of the data suggested that these two effects independently contributed to faster breakthrough times [37]. The current work extends these results and shows that salience could also be conceptualized as the difference between the target stimulus presented to one eye and the CFS mask presented to the other eye, and both salience and task-relevance of color can independently shorten breakthrough times under these conditions. The current study also draws a parallel to research on the mechanisms of (bottom-up) attentional capture, where color contrasts are considered a strong component of stimulus-driven attentional selection [6,7].

In addition to these theoretical considerations, the finding that color contrast determines the strength of interocular suppression also has methodological implications for studies that use CFS to render stimuli invisible. Some studies emphasized the importance of low-level stimulus features in CFS [26,27,36]. However, there is little consistency concerning the choice of stimuli between different studies [38]. Specifically, the variability in the choice of spatial, temporal, and chromatic features of CFS masks is high across studies [27,36,38]. The critical importance of feature similarity between suppressed stimuli and CFS masks entails the risk of theoretically problematic confounds, especially when visually different stimuli are used in different experimental conditions [39]. For example, if a study finds that emotional pictures break suppression faster than neutral pictures, one might be inclined to conclude that emotional stimuli have preferred access to consciousness. However, upon closer examination, one might find that the color distribution in the emotional pictures might be more distinct from the color distribution of the CFS mask. In contrast, the emotionally neutral pictures might share more features with the CFS masks, hence leading to more robust suppression.

Thus, such an effect could be, at least partly, explained by low-level feature differences and might have less to do with the semantic or emotional significance of the pictures. Excluding such confounds is critical but not necessarily an easy endeavor [40]. For example, one way to make sure that differences in breakthrough times are indeed related to emotional valence is to use conditioning procedures to generate arbitrary pairings of visual properties and emotional valence. Using this approach, one study found that colored stimuli that were associated with an electrical shock during a previous fear conditioning phase resulted in shorter breakthrough times compared to probes with a different color that were not associated with the negative experience of an electrical shock [41]. In general, experimental consciousness research needs to take sensory explanations into account in order to make theoretical progress in understanding the extent and limits of unconscious cognition, and gain a better understanding of the factors that accelerate access of a stimulus to conscious awareness [17,18,20].

### 4.2. The Top-Down Effect of Task Relevance on Breakthrough Times

The second major finding of the current study is that color can also shorten breakthrough times in a top-down direction, given a specific task context. If a specific target color was task-relevant, stimuli that matched this task set were reported faster compared to conditions where target color was irrelevant and targets could change color from trial to trial. This observation is closely related to prior research using the CFS paradigm, which showed that stimuli that match visual working memory (VWM) contents enter consciousness faster [42,43]. In these studies, participants were typically instructed to keep an initially presented and visible stimulus in working memory and compare it to a stimulus presented after a delay. Within the delay between presenting the to-be-memorized stimulus and the comparison stimulus, participants were presented with a CFS sequence that rendered a probe stimulus invisible. Importantly, this initially invisible probe stimulus could either match the features held in VWM, or not. A consistent finding was that stimuli that matched the VWM contents escaped interocular suppression faster [42]. The current study illustrates that an explicit instruction to search for a specific color also leads to faster reports of stimuli that match this color. The present findings are also consistent with the results of a recent study where the instruction to search for a target of a particular color shortened breakthrough times of interocularly suppressed stimuli matching that color (cf. Experiment 3 in [10]). The parallels between the present findings and the results from VWM experiments support the view that VWM and top-down guided visual search are closely related processes [44]. Further research could clarify if other features that typically guide attentional selection, such as orientation or shape [9,45], could facilitate reports of interocularly suppressed stimuli. Based on the findings about the relationship of VWM contents and conscious access [42,43,45], setting up an attentional template for specific features could lead to faster perception of the initially invisible stimuli. In this sense, attention could indeed act as a ‘gatekeeper’, or maybe more accurate, as a ‘pathfinder’ to consciousness.

At first glance, the current study suggests that top-down effects could be rather limited in terms of flexibility. Breakthrough times were shorter when participants paid attention to one specific target color, as compared to trials where target color was irrelevant and targets could change color from trial to trial. On the one hand, this is impressive because only one grating was presented in the color-irrelevant conditions, so that no decision was necessary whether it was the correct target object or the incorrect distractor object. Moreover, the target objects could always have only either one or the other color. Suppose participants would be able to search for two different colors in parallel. In that case, they could have easily taken advantage of the two possible target colors, even if color was not explicitly task-relevant. Surprisingly, however, the opposite was the case. Although one could argue that the task itself is objectively easier, since there is no need to decide whether an object has the correct color or not, the target gratings, in fact, remained perceptually suppressed for a longer duration. Related to these considerations, a previous study reported that when the task requires participants to keep two items in VWM, a target that matches either of these VWM contents breaks through CFS faster than a target that does not match any of these memorized items. However, in such two-item conditions, the breakthrough time is generally increased compared to conditions where observers only have to keep one single item in VWM, and the interocularly suppressed target matches this single memorized item [46]. Of note, previous research suggested that differences in the specific details between experimental paradigms (e.g., regarding the stimulus configurations or the task instructions) could be critical determinants of whether performance costs occur in conditions where participants have to keep multiple items in VWM [47].

In visual search experiments, when targets are usually not rendered invisible by CFS, the participants’ performance can also be hampered by the need to search for more than one target color in parallel [48,49,50]. Something similar seems to manifest in the present data, where specifying a single target color increased performance. Conversely, participants did not benefit from knowing that the target could have only one of two possible colors when color was irrelevant for the task. However, whether or not this observation corroborates the view that participants cannot search for more than one color in parallel needs to be clarified in further experiments. Future research should also place scrutiny on specific differences between experimental paradigms (including stimulus configurations and task instructions), as these could be decisive for whether or not searching for two or more items in parallel might or might not entail performance costs relative to conditions where search is focused on one feature expression [47]. In case of the present experiment, participants might have realized quickly that the targets and CFS masks can both change color randomly from trial to trial, which is why they might have thought of it as a dimension that is not helpful for identifying the target. Thus, future research could instruct participants more explicitly to search for one or the other target color among differently colored distractors under CFS conditions and map out the conditions under which more flexible expectations about the target’s appearance might result in similar benefits compared to conditions with one specific target color.

A potentially fruitful future research avenue opens up by asking whether the limitation of top-down guidance to simple templates of one specific feature depends on the level in the visual processing hierarchy at which these features are represented. Interestingly, previous research using the CFS paradigm showed that expectations about the appearance of more “high-level” stimulus categories could also shorten the time that stimuli require to break into consciousness [51,52]. In these experiments, before the start of each trial, an explicit word cue was presented that either matched the perceptually suppressed stimulus or not. Valid expectations regarding the stimulus category consistently accelerated conscious awareness: A picture of a face is recognized faster if preceded by the congruent word cue “face”, whereas it takes longer if it is preceded by the incongruent word cue “house”. An open question is whether such expectancy effects might ultimately result from relatively coarse attentional settings. Following this hypothesis, the word “house”, for example, might tune attention to vertically and horizontally oriented lines. In contrast, the presentation of the word “face” might tune attention to more curved shapes, or a typical facial configuration of two eyes and a mouth. It could also be that attention can be tuned to different feature dimensions in parallel. For example, when searching for faces, not only shape features but also typical colors commonly seen in faces could be part of the top-down attentional tuning for faces. Future research should clarify if color-guided attention can work in parallel with feature templates from other dimensions, such as orientation or shape, for establishing more effective attentional guidance and facilitate entry to consciousness.

### 4.3. Limitations

Breakthrough times in CFS are often interpreted as a proxy for conscious access but sometimes also as a measure of unconscious processing [20]. For example, some studies assumed that if certain stimuli have shorter breakthrough times, they must have received privileged unconscious processing. To back up this idea, studies often included visible control conditions, in which targets are superimposed on the CFS masks and presented to the same eye, so that no interocular suppression occurs. In case the experimental effects occur only under CFS but not under visible control conditions, previous studies sometimes concluded that the effects reflect unconscious processing. This logic and procedure can be criticized for various reasons. A general problem is that non-dichoptic control conditions are visually distinct and easily recognizable and therefore might not be well suited as control conditions for CFS experiments [20]. This was one reason why the current study sought alternative comparison conditions and manipulated the strength of suppression via the targets’ spatial frequency while keeping the dichoptic presentation mode constant. Even though this manipulation achieved the desired effect of a significantly reduced efficacy of CFS, it is not possible to draw definite conclusions about whether effects of the color-related manipulations reflected differences in unconscious processing of the initially suppressed stimuli.

A common criticism of the “breaking CFS” paradigm is that breakthrough times may not only reflect differences in perceptual sensitivity or discriminability [53]. Participants may also take more or less time to respond, depending on how conservative or liberal they set their response criterion. Although the analysis of error rates conducted in the current study does not indicate that a speed-accuracy tradeoff could explain the experimental effects in breakthrough times, it is not possible to make a definitive judgment about whether the observed effects reflect only differences in perceptual sensitivity. Signal detection theory [54], as the default framework for separating sensitivity and response bias in perceptual tasks, is not readily applicable to the analysis of breakthrough times. This is because its measures are derived from hit and false alarm rates rather than response times. Still, there is hope that breakthrough time effects do not only reflect differences in response criteria, because research has shown that when perceptual sensitivity is measured directly (e.g., using other masking techniques or with fixed durations of CFS trials), the qualitative pattern of results tends to converge with breakthrough time effects in CFS experiments [51,53].

Finally, caution is also warranted concerning the involvement of selective attention processes in the present effects. Based on previous research on attention, the present study assumed that color could attract attention through (bottom-up) chromatic contrast or guide target search by focusing (top-down) on a specific target color. The involvement of spatial attention seems plausible since the target stimuli were shown at peripheral locations, and the target’s location varied randomly from trial to trial. Also, the CFS masks could act as distractor stimuli that complicate the search for the target if they are similar to the searched target stimulus. Although the current findings, as discussed in the previous sections, have many parallels to research on VWM and attention, there are also clear divergences to the visual search paradigm that dominates research on selective attention. First and foremost, the stimuli presented in visual search experiments are usually not presented under dichoptic conditions. Furthermore, the spatial overlap of relevant target stimuli and irrelevant stimuli (here: the CFS masks) is atypical for visual search experiments since one position is usually occupied by either a target or a distractor. Thus, the current experiment does not provide unequivocal evidence that the effects are due to selective visual attention. Moreover, concerning color-related effects on attentional orienting, the line between unconscious and conscious processing is often not so clear. For example, previous research showed that despite the conscious knowledge of a specific target color, a grouping of similar distractors (which presumably takes place unconsciously) can facilitate target search [55]. Thus, further research is necessary to understand, first, if selective attention is responsible for the effects observed in the present study, and second, whether these attention processes occur consciously or unconsciously.

## 5. Conclusions

The present study found that color can facilitate reports of LSF grating orientations that are difficult to discriminate due to interocular suppression by CFS. First, breakthrough times were shortened when targets and CFS masks had different colors, which probably reflected a sensory (bottom-up) benefit of increased target salience. Second, breakthrough times were also shortened when a specific target color was relevant for the task, which perhaps reflected a task-related (top-down) benefit. The two color-related effects occurred in parallel and did not interact with each other. The relation of these results to the previous literature and practical implications for research using CFS to suppress stimuli from awareness were discussed.

## Figures and Tables

**Table 1:** Means and standard errors (in parentheses) for breakthrough time and error rate in the eight experimental conditions of the present study.

	LSF (2 cpd)		HSF (4 cpd)	
	Different	Same	Different	Same
*Breakthrough time (ms)*				
Color relevant	2675 (216.1)	3117 (257.5)	1778 (143.2)	1738 (137.9)
Color irrelevant	3209 (312.2)	3832 (344.2)	1801 (201.1)	1731 (190.1)
*Error Rate (%)*				
Color relevant	3.05 (0.62)	4.35 (0.84)	2.76 (0.41)	3.78 (0.54)
Color irrelevant	4.59 (1.04)	4.32 (0.95)	2.53 (0.57)	2.21 (0.42)
*Error Rate (arcsine transform)*				
Color relevant	0.13 (0.02)	0.18 (0.02)	0.14 (0.01)	0.17 (0.01)
Color irrelevant	0.17 (0.02)	0.15 (0.02)	0.13 (0.02)	0.11 (0.02)

## Data Availability

The data presented in this study are openly available through the Open Science Framework at https://osf.io/mb6da/, uploaded on 15 January 2021.

## Supplementary Materials

The following are available online at https://www.mdpi.com/2411-5150/5/1/13/s1, Analysis S1: Analysis of normalized breakthrough times, Analysis S2: Exclusion of participants with very short or long breakthrough times.

## Abbreviations

The following abbreviations are used in this manuscript:

BR	Binocular Rivalry
CFS	Continuous Flash Suppression
cpd	Cycles per degree of visual angle
CRT	Cathode Ray Tube
HSF	High Spatial Frequency
LSF	Low Spatial Frequency
SF	Spatial Frequency
VWM	Visual Working Memory
