# Supplementary material for: Color Can Shorten Breakthrough Times in Continuous Flash Suppression through Increased Salience and Task Relevance
Source: Vision (Basel). 2021 Mar 16;5(1):13. doi: 10.3390/vision5010013 (PMC8006049; doi:10.3390/vision5010013)
Supplement: Supplementary file 1 [file vision-05-00013-s001.pdf]

Supplementary material:

Color can shorten breakthrough times in continuous flash suppression through increased salience and task relevance

Christian Valuch

S1. Analysis of normalized breakthrough times

The difference in color-related effects between LSF and HSF targets could be partly due to the generally much longer breakthrough times in LSF conditions. To allow a fair comparison in such situations, a simple normalization method was proposed in previous work [1], where the breakthrough time in each trial is set in relation to the average breakthrough time of the respective experimental condition. Here, this was done by dividing each trial's breakthrough time by the mean breakthrough time from all trials with this target SF. The statistical analysis was then repeated on the mean normalized breakthrough times.

This control analysis resulted in a main effect of Target/CFS color, $F(1, 39) = 14.7$, $p < 0.001$, $\eta^2_p = 0.27$, with shorter breakthrough times when targets and CFS masks had different colors ($M = 0.98$, $SE = 0.01$) compared to when they had the same color ($M = 1.02$, $SE = 0.01$). More importantly, both interactions of the main analysis were replicated using this alternative analysis approach. First, the interaction of Target SF × Target/CFS color was significant, $F(1, 39) = 59.1$, $p < 0.001$, $\eta^2_p = 0.60$. For LSF targets, normalized breakthrough times were shorter when targets and CFS masks had different colors ($M = 0.94$, $SE = 0.01$) compared to when they had the same color ($M = 1.06$, $SE = 0.01$), $t(39) = –6.1$, $p < 0.001$. For HSF gratings, identical to the main analysis, the opposite tendency was observed, namely slightly longer breakthrough times when targets and CFS masks had different colors ($M = 1.02$, $SE = 0.005$) compared to when they had the same color ($M = 0.99$, $SE = 0.005$), $t(39) = 3.2$, $p = 0.003$. Second, the interaction of Target SF × Color relevance, was also significant, $F(1, 39) = 16.5$, $p < 0.001$, $\eta^2_p = 0.30$. For LSF gratings, normalized breakthrough times were significantly shorter when target color was task-relevant ($M = 0.93$, $SE = 0.02$) compared to when it was irrelevant ($M = 1.07$, $SE = 0.02$), $t(39) = –3.0$, $p = 0.005$. For HSF gratings, the normalized breakthrough times were not different when target color was relevant ($M = 1.00$, $SE = 0.02$) compared to when it was irrelevant ($M = 1.00$, $SE = 0.02$), $t(39) = 0.02$, $p = 0.983$. No other effects were statistically significant in this analysis. Thus, this control analysis replicated both interactions from the main analysis, which excludes the possibility that the absence of facilitating effects of color salience and task relevance for HSF gratings could be a statistical artifact due to the absolute difference in breakthrough times between SF conditions.

S2. Exclusion of participants with very short or long breakthrough times

Three participants had a median breakthrough time that fell in the range before the target reached its final contrast in at least one experimental condition. Four other participants had a medium breakthrough time that fell in the range when the CFS masks had already faded out in at least one condition. Thus, these seven participants could be considered outliers because for them, interocular suppression was either ineffective or unusually strong compared to most participants. Therefore, an additional control analysis was performed to check whether the main results remain valid if these peculiar participants are excluded from the analysis.

This control analysis confirmed the main effect of target SF, $F(1, 32) = 77.7$, $p < 0.001$, $\eta^2_p = 0.71$, with longer breakthrough times for the LSF targets ($M = 3023$ ms, $SE = 197.4$) compared to the HSF targets ($M = 1606$ ms, $SE = 78.2$). There was also a main effect of target/CFS color, $F(1, 32) = 17.2$, $p < 0.001$, $\eta^2_p = 0.35$, with shorter breakthrough times when the targets and the CFS masks had different colors ($M = 2189$ ms, $SE = 122.0$) compared to when they had the same color ($M = 2441$ ms, $SE = 138.2$). More importantly, this control analysis also replicated both interactions from the main analysis. First, the

interaction of Target SF × Target/CFS color was significant, $F(1, 32) = 28.3$, $p < 0.001$, $\eta^2_p = 0.47$. For LSF targets, breakthrough times were shorter when targets and CFS masks had different colors ($M = 2751$ ms, $SE = 185.2$) compared to when they had the same color ($M = 3295$ ms, $SE = 223.7$), $t(32) = -4.8$, $p < 0.001$. Again, for HSF gratings, the opposite tendency was observed, namely slightly longer breakthrough times when targets and CFS masks had different colors ($M = 1626$ ms, $SE = 84.2$) compared to when they had the same color ($M = 1587$ ms, $SE = 73.5$). However, this tendency was not statistically significant in the reduced dataset, $t(32) = 1.7$, $p = 0.105$. Thus, the significant difference in the main analysis should be interpreted with caution as it does not seem to be particularly robust and might be driven by participants with more extreme breakthrough times. Second, the interaction of Target SF × Color relevance, was also significant, $F(1, 32) = 6.8$, $p = 0.014$, $\eta^2_p = 0.18$. For LSF targets, breakthrough times were significantly shorter when target color was task-relevant ($M = 2764$ ms, $SE = 191.4$) compared to when target color was irrelevant ($M = 3283$ ms, $SE = 265.9$), $t(32) = -2.1$, $p = 0.040$. For HSF targets, breakthrough times were not different when target color was relevant ($M = 1631$ ms, $SE = 70.7$) compared to when it was irrelevant ($M = 1581$ ms, $SE = 95.6$), $t(32) = 0.8$, $p = 0.428$. No other effects were statistically significant in the analysis of the reduced dataset. Thus, this control analysis replicated the two critical interactions in the main analysis, which suggests that the core findings are not an artifact of the presence of participants with particularly long or short breakthrough times in some of the conditions.

References

1. Gayet, S.; Stein, T. Between-subject variability in the breaking continuous flash suppression paradigm: Potential causes, consequences, and solutions. *Front Psychol* **2017**, *8*, 437.
